# Novel Materials for Additive Manufacturing

**DOI:** 10.3390/ma19081533

**Published:** 2026-04-11

**Authors:** Joan-Josep Suñol

**Affiliations:** Department Physics, P2, EPS, Campus Montilivi s/m, University of Girona, 17003 Girona, Spain; joanjosep.sunyol@udg.edu

In recent decades, additive manufacturing (AM) techniques have been used to produce all kinds of materials, initially polymers. It is a continuously growing field of research with ongoing technical improvements and applications to new types of materials. This Special Issue is dedicated to additive manufacturing, which traditionally was static 3D printing [1,2]. However, a new approach is 4D printing, which adds the dimension of time to 3D printing, enabling objects to modify shape, enhance properties, or improve functionality autonomously after production [3]. The additive manufacturing process usually involves the fabrication of three-dimensional parts directly from CAD models by adding materials layer by layer. Thus, it is able to build components with complex geometries. It is being applied in the processing of all types of materials, whether traditional (ceramics, metals, polymers, composites) [4,5,6,7], biological [8], or food-related [9]. The objective of this Special Issue was to collect articles concerning the applications of different additive manufacturing techniques, such as stereolithography, digital light processing, selective laser sintering/melting, electron beam melting, fusion deposition modeling, multijet/polyjet 3D printing, direct injection writing, and laminated object manufacturing. A set of nine articles have been published. Therefore, not all processes and materials have been covered. However, taken together, they provide an initial overview of several areas of ongoing research. The following is a summary of the main results and conclusions of the published works.

Several articles are associated with the development of parts by material extrusion (MEX) AM techniques. One of these techniques, fused deposition modeling (FDM), allows for the generation of prototypes and manufactured parts by depositing melted filament material layer by layer according to a pre-determined path [10]. In one paper, the authors fabricate orthopedic plates by varying the infill levels. It is known that the introduction of additive manufacturing techniques has led to the creation of new design implants with complex internal architectures. The articles analyze the influence of the infill density and the internal holes on the fatigue crack rock resistance using a numerical-based model incorporating honeycomb infill structures. The spatial distribution of internal holes (relative to the initial crack location) influences fatigue significantly. The infill density (except for 90% infill) is a minor effect. Thus, the distribution of internal holes and the customization of the internal geometry have the potential to increase fatigue life. Another manuscript examining material extrusion is based on the design of inserts in injection molding (IM), offering advantages in product development and customization. MEX is applied due to its affordability and recyclable materials. Regarding applications, MEX fabricates filled filaments with optimized thermal and mechanical responses. LDPE and PP were selected as thermoplastics, and simulations of temperature and cavity pressure were performed. It should be remarked that all inserts retained their structural integrity throughout ten injection molding cycles. This work concludes that MEX-based AM using filament feedstock is a viable alternative method for producing injection molding inserts (in small production batches).

With respect to fused deposition modeling, also known as fused deposition fabrication (FFF), another study proposes an adapted methodology based on the Average Strain Energy Density (ASED) criterion to predict fracture loads in 3D-printed polymers (FFF) with nonlinear behavior. Tests were performed on two different materials with different raster orientations and notch radii, specifically acrylonitrile–styrene–acrylate (ASA) and carbon fiber-reinforced ASA (ASA-CF10) specimens. This study validates the use of the calibrated ASED criterion to assess the structural integrity of 3D-printed components and suggests extending the analysis to other polymeric materials. It should be noted that the selected materials do not exhibit perfectly linear behavior. Therefore, proper calibration is essential to achieve high accuracy. The optimization of processing parameters is necessary to improve the final quality of the produced parts.

Continuous filament fabrication (CFF) involves the deposition of continuous carbon fiber filaments within a thermoplastic material matrix [11]. This process combines the properties of carbon (strength and light weight) with those of thermoplastic materials. The main objective is to fabricate robust and lightweight parts as sandwich structures. A study analyzes the in-plane compression behavior of 3D-printed sandwich panels with two cell geometries and two material configurations. These panels are candidates for applications in lightweight structures. The cell geometries were hexagonal and re-entrant, and two material configurations were evaluated: Onyx (a nylon-based composite) and a reinforced configuration with carbon fibers (CCF). The analysis of the load-bearing capacity determined that the reinforced panels have a 35% higher load-bearing capacity (but fail with a 55% lower displacement) than the unreinforced panels. The reinforced re-entrant panels exhibited more flexible behavior and superior energy absorption. Concerning the experiments made under compression, the reinforced panels showed greater stiffness and load-bearing capacity and a greater variability in energy absorption. One of the main conclusions is that the cell geometry and the inclusion of continuous carbon fibers have a significant impact on the structural performance of the panels.

Another AM technique is laser powder bed deposition (LPBF). This technique is applied to fabricate parts by selectively melting layers of metal powder using a laser [12]. In a study, the viability of high-energy LPBF processing for WE43 magnesium alloys was analyzed, detecting enhanced mechanical response and corrosion resistance. Thus, this material is a candidate for biomedical and structural applications. Microstructural analysis revealed the presence of refined grains and the presence of rare-earth RE-rich intermetallic particles: regarding homogeneity, the microhardness increased with height due to thermal gradients. Electrochemical testing was carried out in a highly aggressive medium in 3.5 wt.% NaCl solution. The corrosion of samples with density values below 99% is conditioned by the porosity. Likewise, at higher values, the corrosion evolution is linked to the microstructure of the samples.

Selective electron beam melting (SEBM) is a powder bed fusion additive manufacturing technology that uses a high-energy electron beam to melt metal powder layer by layer [13]. One study applied this technique to investigate the variant-specific deformation mechanisms of a melted TA15 titanium alloy (Ti-6.5Al-2Zr-1Mo-1V) treated with hot isostatic pressing (HIP). The main objectives were to understand how the orientation of the variants influences the plastic behavior of the α phase and to improve the texture and performance-oriented design of these alloys. The α variants exhibit different slip mechanisms depending on their orientation. The α4–α6 variants primarily activate prismatic slip, while the α1–α3 variants depend on other slip systems or twinning. Activated variants facilitate stress relaxation and improve strain homogeneity. The combination of two deformation mechanisms (slip and twinning) contributes to the simultaneous improvement of the ductility and strength of the TA15 alloy.

For the additive manufacturing process, the rheology of the inks and mixtures should be optimized to favor flowability during the fabrication of the specimens. The rheology determines the material flow, extrudate shape retention, and layer adhesion [14]. Critical parameters associated with printability are viscosity, shear-thinning behavior, and yield stress. The published study applies a full-factorial approach to systematically determine the coupled effects of polymer concentration and pH on the rheological properties of ETD2020. Carbopol^®^ Carbomers (Lubrizol, Wickliffe, OH, USA) are frequently used as a support matrix in embedded printing due to their high transparency, biocompatibility, and tunable rheological profile. The main objective is to define robust formulation regimes that are tailored for embedded printing. The best fit is provided by the Herschel–Bulkley model for nearly all formulations. As expected, the local heterogeneities negatively affect strand definition, surface quality, and material fusion during embedded printing. In a study published in this Special Issue, the modified multifunctional additive, boron polyol complex with boron nitride BPC–BN, was used for the functional modification of epoxy resin. It is known that fire retardancy and thermal management improvements in epoxy resins are candidates for applications in electronics for IoT and 5G devices. The study introduces a method to improve fire retardancy and the thermal properties of epoxy resins by incorporating boron nitride nanoparticles (with boron polyol complex). This creates a synergistic effect between the polyelectrolytes and the BN, and an ionanofluid is produced. The main results show enhancement of the material properties due to the chemical interaction between the constituent additives and the epoxy polymer chain. The remarkable fire retardancy is due to the synergistic effect of the BN nanoparticles and BPC.

Additive manufacturing processes are complex, making the use of simulation methods and the influence of processing conditions on the characteristics of the final product a topic of interest [15]. Another area of interest is the development of artificial intelligence methods, such as those related to artificial intelligence and machine learning, for selecting compositions and processing conditions [16]. The last contribution explores the application of machine learning techniques in structural optimization in AM metamaterials. Their flexibility, adaptability, and efficiency in solving a wide range of problems should be highlighted. The engineering application of metamaterial design used a convolutional neural network together with the greedy algorithm. This combination enables the optimization of the design and development of bi-materials (with negative Poisson’s ratios) made from a hard and a soft material, intended for fabrication through additive manufacturing. It is mandatory to optimize the model construction, enhance the model training, and proceed to model validation. In this case, the optimal solution showed a high degree of similarity with known cells in the scientific literature, referred to as tetra-anti-chiral cells.

Finally, we would like to thank all the authors, the reviewers, and the publishing house’s technical staff for their participation in this Special Issue. It is worth noting that the development of new materials through additive manufacturing is a continuously growing field.
